# Boosting antenatal care attendance and number of hospital deliveries among pregnant women in rural communities: a community initiative in Ghana based on mobile phones applications and portable ultrasound scans

**DOI:** 10.1186/s12884-016-0888-x

**Published:** 2016-06-14

**Authors:** Benjamin Amoah, Evelyn A. Anto, Prince K. Osei, Kojo Pieterson, Alessandro Crimi

**Affiliations:** ETH Zurich, Zurich, Switzerland; University of Ghana, Accra, Ghana; African Institute for Mathematical Sciences, P.O. Box DL 676, Cape Coast, Ghana; Korle Bu Teaching Hospital, Accra, Ghana; Istituto Italiano di Tecnologia, Genoa, Italy

**Keywords:** Ghana, Antenatal care, Pregnancy, Mobile phones, Portable ultrasound, Skilled attendants

## Abstract

**Background:**

The World Health Organization has recommended at least four antenatal care (ANC) visits and skilled attendants at birth. Most pregnant women in rural communities in low-income countries do not achieve the minimum recommended visits and deliver without skilled attendants. With the aim of increasing number of ANC visits, reducing home deliveries, and supplementing care given by ANC clinics, a proposed system based on low-cost mobile phones and portable ultrasound scan machines was piloted.

**Methods:**

A sample of 323 pregnant women from four rural communities in the Central Region of Ghana were followed within a 11-month project. In each community, at least one health worker was trained and equipped with a mobile phone to promote ANC and hospital deliveries in her own community. If women cannot attend ANC, technicians acquired scans by using portable ultrasound machines in her community directly and sent them almost in real time to be analyzed by a gynecologist in an urban hospital. A preliminary survey to assess ANC status preceding the pilot study was conducted. During this, one hundred women who had had pregnancies within five years prior to the study were interviewed.

**Results:**

The preliminary survey showed that women who attended ANC were less likely to have a miscarriage and more likely to have delivery at hospital or clinic than those who did not, and women who attained at least four ANC visits were less likely to practice self-medication. Among the women involved in the project, 40 gave birth during the period of observation. The proposed prenatal care approach showed that 62.5 % of pregnant women who gave birth during the observation period included in the project (*n*=40) had their labor attended in clinics or hospitals as against 37.5 % among the cases reported in the pre-survey. One case of ectopic and two cases of breech pregnancies were detected during the pilot through the proposed approach, and appropriate medical interventions were sought.

**Conclusion:**

Our results show that the proposed prenatal care approach can make quality ANC accessible in rural communities where pregnant women have not been able to access proper ANC.

## Background

In 2013, globally, an estimated 800 women died from pregnancy and childbirth-related complications each day. Low-income countries account for 99 % of these deaths, with the sub-Saharan Africa region alone accounting for 62 % [1]. The adult lifetime risk of maternal mortality in women from sub-Saharan Africa was 1 in 38, in sharp contrast to 1 in 3700 among women in high income countries [1]. Maternal mortality in Ghana in 2013 was estimated at 380 (per 100,000 live births), a figure which is far above the 185 targeted in Millennium Development Goal (MDG) Five [1, 2]. Despite the progresses made, Ghana did not achieve the MDG Five, especially in rural isolated communities.

Most maternal deaths are due to obstetric complications which can be prevented or detected and managed if pregnant women get early access to available intervention programs. Infections, malaria, anemia, and antepartum hemorrhage among other causes of maternal death can be treated, whereas other causes such as pre-eclampsia and eclampsia can be managed during ANC [3]. The attendance of birth by skilled health professionals presents opportunities for preventing some maternal and child deaths as it ensures clean and safe delivery practices. Clean deliveries prevent puerperal sepsis, a major cause of maternal deaths [4]. Skilled attendants also ensure effective handling of labor, delivery and postpartum complications such as obstructed labor, breech birth, postpartum hemorrhage, retained placenta, and other emergencies [5]. Ultrasound-based analysis is the most accurate method to assess breech pregnancies [6], and the key to diagnose ectopic pregnancies and other dangerous pathologies [7].

Due to the shortage in medical staff, facility and equipment in rural communities, women living in rural and poorer communities are more likely to die from pregnancy and childbirth complications than those who live in urban communities [8]. In rural areas the distance to modern ANC providers can be considerable. One out of nine women have to travel more than 15 km to reach medical care, thus these women might restrain their ANC [9]. Unsurprisingly, fewer ANC visits in rural areas are accompanied by more infant mortalities per 1000 live births (rural: 15, overall: 10) [10].

One way to improve the ANC management is to enable access to remote medical expertise and technology for local health-care facilities by equipping them with advanced telecommunications services for telemedicine purposes, and then to appropriately use remote expertises and technologies. Despite the uneven infrastructures across regions including the landline networks (and therefore Internet), mobile phone penetration has increased exponentially. Africa has now more than 650 million mobile phone subscribers. Since 2000, the mobile phone market has grown 40-fold [11].

Given these large existing mobile infrastructures, in the last years, there have been a plethora of promising mobile phone applications with several aims. RapidSMS was introduced in Malawi by UNICEF’s innovation unit to allow health workers to enter child’s data and receive instant alerts on nutritional status [12]. Afterwards it has been used in numerous projects including ANC [13]. Similar technologies are FrontlineSMS and its derived MedicMobile [14], and CommCare-HQ (Dimagi Inc., MA USA). Commcare-HQ mobile application has been used so far in several projects for data collection and as an educational tool. It is not limited to short messaging service (SMS) and can include audio, image, and video prompts within the application itself. For a review on projects using the Commcare-HQ platform, the reader is addressed to [15].

Community health workers (CHWs) are volunteer members from rural communities who fulfill the role of health promoters and home-based providers. They are not professional medical doctors or nurses, but they are trained to carry out simple but effective tasks. CHWs have been shown to be particularly effective in supporting maternal care and helping reduce newborn mortality. A study in Bangladesh showed a 34 % decrease of neonatal mortality in cases supported by CHW intervention against unsupported contexts [16]. Similarly, another study in India showed a 54 % reduction of neonatal mortality in cases supported by CHWs [17]. A case-control study called *“Wired-Mother”* showed that a CHW intervention using an SMS approach with mobile phones significantly increased the proportion of women receiving the recommended four ANC visits during pregnancy [18].

The objectives of the study were to increase ANC attendance, promote early ANC attendance and skilled birth attendance, and prevent pregnancy complications. As shown in the pilot, this could be reached by the proposed settings combining CHWs and portable ultrasound acquisitions. The following section illustrate a survey conducted preliminary to the study to assess the baseline status of ANC in the communities under study. The remainder of the manuscript describes a pilot project in Ghana using CHWs endowed with mobile phones and trained technicians acquiring ultrasound scans directly in the community. To the best of our knowledge, no previous study has been conducted by integrating ultrasound scans, mobile technologies and community initiative.

## Methods

This section comprises the survey pre-study used to assess the baseline status of the communities, and the used approach for the pilot study. The target location of the pilot was the Central Region of Ghana. The Central Region has 13 administrative districts, an estimated population of almost two million people and an annual population growth rate of 2.1 %. The region is the second most densely populated in the country, with the 63 % of its population living in rural areas. It has in all 193 health facilities comprising 77 public, 100 private, and 16 missions. However, most of these institutions are located in the Cape Coast municipality, leaving some of the remaining 12 administrative districts relatively disadvantaged.

### Survey preceding roll out of pilot ANC system

#### Data collection and analysis

Women who had had pregnancies within five years preceding the study were interviewed. The women were from four rural communities of the Mfantsiman, the Ekumfi and the Ajumako-Enyan-Essiam district in the Central Region of Ghana. The communities are Ekumfi-Asafa, Ekumfi-Edumafa, Biriwa and Ankokrom-Owomasi. These are the same communities where the pilot was carried out.

The communities were chosen among those between the city of Cape Coast and Accra to facilitate the trips of the ultrasound technicians. Moreover each community represented a specific condition:

- in proximity of a clinic and well connected with the major cities (Biriwa),
- in proximity of a clinic but not well connected to major cities (Ekumfi-Edumafa),
- located at 5 km from a clinic and isolated (Ekumfi-Asafa),
- located at more than 10 km from a clinic and isolated (Ankokrom-Owomasi).

With each community representing a stratum, a snowball sampling technique was used to reach respondents in each community. In all, one hundred women were interviewed (although the aim was to collect twenty-five for each community, there were more interviews from some communities rather the others). Data were collected using a structured questionnaire and in-depth interviews. In the event that a woman had had more than one pregnancy in the specified five years, she was allowed to choose which one she wanted to be interviewed about before the interview commenced. Whenever it was appropriate, secondary data were extracted from ANC booklets of participants. If a pregnant woman took any drug (apart from pain killers, e.g. taking paracetamol as first aid) without the prescription of a qualified practitioner, and in an attempt to treat any of the complications listed in Table 1, it was considered as self-medication. Data from the completed questionnaires were entered into and analyzed with SPSS version 16.0 (IBM, New York, USA). Statistically significant relationships among the collected information were sought, and a *p*-value less or equal to 0.05 was considered to indicate a significant value. The quantitative information was complemented with qualitative data obtained through the interviews. Moreover, a brief focus group discussion among the CHWs was conducted during the training session for CHWs to identify further relevant elements. After revision of notes, the transcripts were typed and coded by using the software NVivo 10 (QSR International, Melbourne, Australia). The group discussion notes and the interviews were thus analyzed by using qualitative content analysis [19]. The results of the survey are in the first part of the section Results.

**Table 1:** Complications reported in the survey women experienced during pregnancy (*n*=100, categories are not mutually exclusive)

Complication	Frequency
Persistent dizziness or headaches	45
Heart palpitations	36
Blurred vision	34
Fever	27
Severe nausea and vomiting	26
Recurring pain in abdomen or chest	26
Swelling of face and/or leg	17
Vaginal bleeding	12
Miscarriage	12
Decreased fetal movement	11
Stillbirth	7
Severe diarrhea	6
Accident or impact	6
Fainting	4
Other	6

### The pilot project

**Fig. 1:**
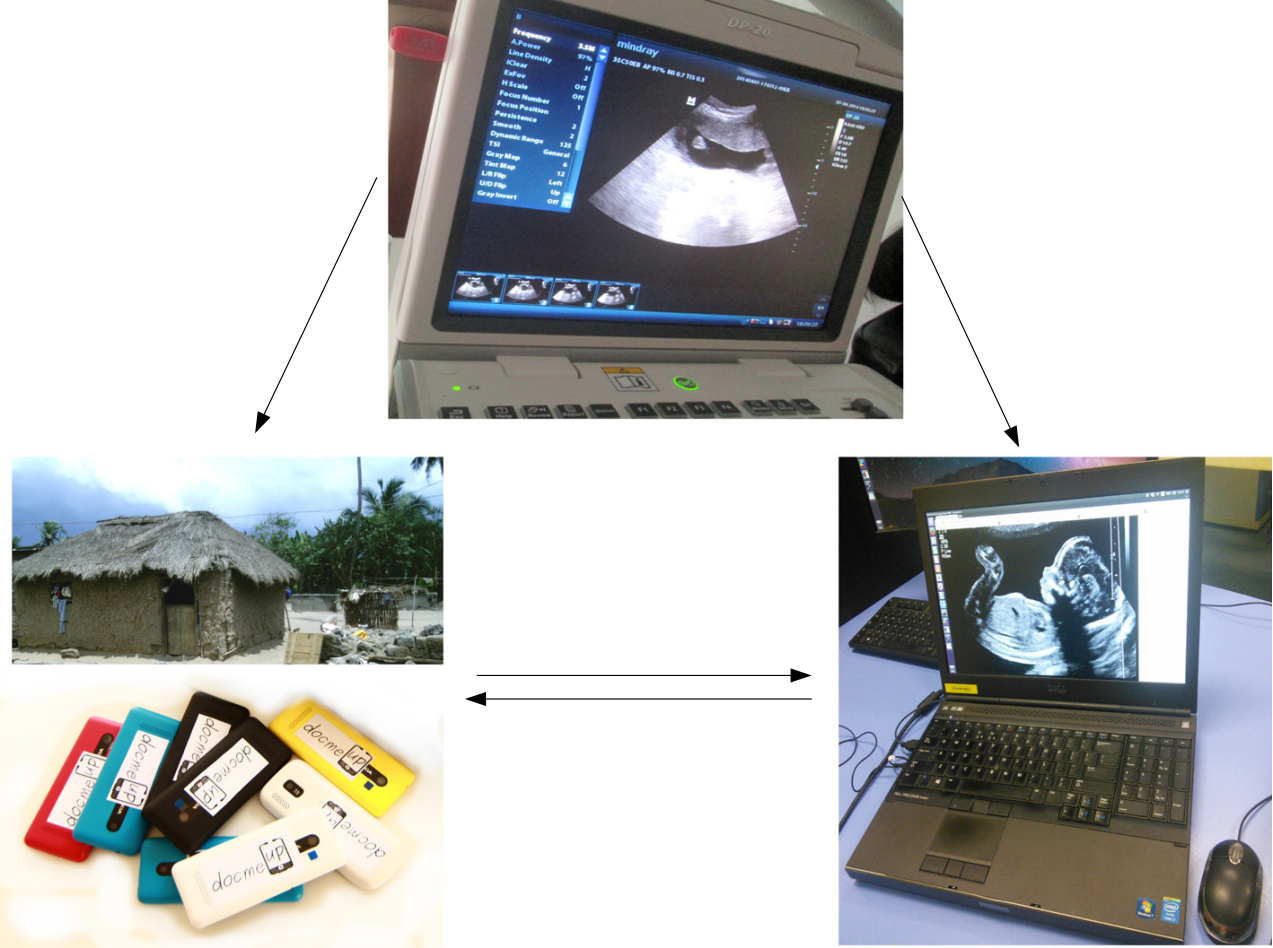
The main components of the system: the CHW (represented by mobile phones and the mud-house), a technician acquiring ultrasound scans (the author BA), and the online platform. All people appearing in the image provided consent for the image to be published

The project comprised three different tiers which are depicted in Fig. 1: A community initiative, an online management system and a remote ultrasound imaging approach. Several pregnant women were followed in their communities by CHWs and ultrasound technicians, which tracked the pregnancy evolutions through low-cost mobile phones and a portable ultrasound scan machine. A centralized system recorded all information coming from the mobile phones of CHWs within the rural communities, giving also the possibility to report danger signs. The CHWs were supposed to support all pregnant women at all trimesters. However, the inclusion criterion for the monitoring part of the project was that the pregnancy should be detected during the first trimester to assure that results of the project are given by the positive impact of the project.

#### Communities initiative

The communities initiative was the practical channel through which the project was carried out. In this initiative, CHWs as well as technicians interacted with pregnant women in the communities. For each community, at least a volunteer was selected during a public assembly and later trained to become a CHW. The CHWs were not expected to be formal health professionals, but literate female members of the communities in high consideration by the stakeholders of the community. Other pre-requisites were fluency in English language and a senior high school certificate. The CHWs were trained and equipped with low-cost mobile phones (Nokia ASHA 206). Their duties were to sensitize community members about the need to attend ANC and have a hospital delivery, carry out pregnancy test for women with suspected pregnancies in order to have ANC initiated early, register new pregnancies, send information to a centralized online platform by using the phone application, frequently visit all pregnant women they have registered so as to report all danger signs and educate pregnant women on important topics such as family planning, personal hygiene, nutrition, etc. All the communications to the community are sent through the CHWs and not to the pregnant women directly.

The CHWs were supposed to check fortnightly the status of the registered women, recommending at least 4 ANC visits within the pregnancy and tracking whether they were effectively attending the visits. Those who were not following this behavior were notified to the ultrasound technicians troughs the system for monthly ultrasound scans. All pregnant women who allowed the use of their data in anonymous manner were involved into the initiative. The follow-ups including the delivery at health facilities or at home were assessed and reported by CHWs who were following the pregnant women even after the delivery. During the pilot, a total number of 5 CHWs were involved. One in each community, except Ekumfi-Edumafa where due to its highly fragmented geographic structure, the community was subdivided into “east” and “west” requiring two CHWs. Some women did not want to be enrolled in the study due to pressure of the husband or the local herbalist.

#### Online platform and mobile phone application

**Fig. 2:**
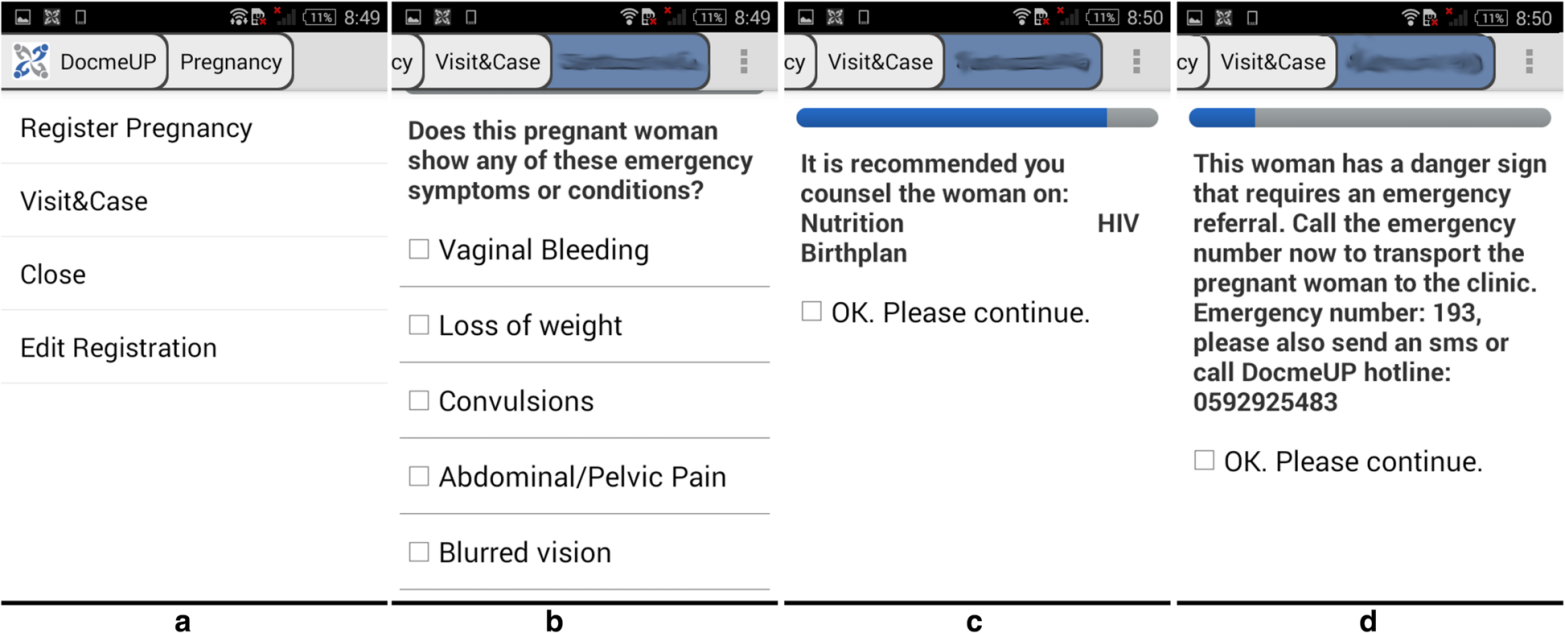
Some representative screen-shots of the mobile phone application. From left to right, screen-shot-(**a**) depicts the main menu, screen-shot-(**b**) highlights some of the possible symptoms which can be reported by the CHW, screen-shot-(**c**) and -**d** depict possible suggestions that can be given to the CHW, as a recommended counseling or the urge of an emergency referral respectively

The information technology (IT) infrastructure was enhanced by a general packet radio service (GPRS) based telecommunication system, accessible by affordable mobile phones (one given to each CHW). In practice, the system was divided into server and client side. The server side comprised a password-protected web user interface, which gave access to aggregated and individual data and enabled tracking the history of individual patients as well as automatic report generation. This latter web interface could give an overview including individual and aggregated reports, statistics, log of reminders and activity within the community. The client side was given by a mobile phone application called DocmeUP, which was created for Java and Android system using Commcare-HQ. DocmeUP comprised two main modules as shown in Fig. 2(a) *“Register pregnancy”* and *“Visit & case”*. *“Register pregnancy”* allowed the CHW to enter a new pregnant woman’s data into the system, with her personal data including the current *estimated delivery date* (EDD), or the fact that the current pregnancy is the first. Two other options enabled data update (*“Edit a registration”*), and to notify that a woman has left the community or died (*“Close”*).

The module *”Visit & case”* allowed CHWs to report danger sign(s) experienced for previously registered women and immediately receive instruction concerning how to handle such complication. Figure 2(b) shows some of the emergency symptoms or conditions. Depending on the information the CHW has entered, one of these three possibilities will be suggested:

- recommend counseling,
- require assistance within 24 hours,
- require emergency referral immediately.

The topic for the suggested counsel -as shown in Fig. 2(c)- depended on the data entered and on other information previously given about the woman. Alternatively, the CHW could be directed to refer the woman to a clinic either within 24 hours or immediately as shown in Fig. 2(d). At the same time that the instruction was given to the CHW, the online platform records the distress signal in the database.

#### Remote ultrasound imaging

An objective of the project was to ensure each pregnant women involved attended the examination sessions conducted by a gynecologist. However, when this was not possible, a trained technician visited the community and acquired some scans which, in quasi-real-time, were sent to a gynecologist in urban hospitals for analysis and immediate feedback. In this way, data from ultrasound scans were acquired directly in the rural areas without the physical presence of a doctor, and immediately sent to be investigated in urban clinics by gynecologists. In this pilot study, only one portable ultrasound (DP-20, Mindray, Shenzen, China) with a 3-5 MHz convex transducer was used. An examination was conducted on as much as possible women each month, mainly caring for the women who did not appear attending the expected number of ANC visits. Having only one possibility per month for each community, not all women in the project were scanned. After the acquisition of the scan data, the off-line examination is performed remotely by the expert gynecologist.

During the examination, a general foetal survey was done and biometric features [20] were measured; these helped to get an accurate due date of delivery and the status of the child. More specifically, the early first trimester scans were taken for accurate dating of pregnancy using as biometric features the mean sac diameter, crown rump length, biparietal diameters. The CHW had an important role related to the ultrasound acquisition because they were educated to address pregnant women mainly in the first trimester for scan, where gestational age and EDD can be more accurately determined. For the early scans especially those before 6 weeks, the cardiac activity were not visualized. Such pregnant women were rescheduled after some weeks depending on when they reported for fetal viability confirmation from cardiac activity presence. Subsequently, scans were done for fetal growth monitoring especially using the femur length (FL) and abdominal circumference (AC). In the events of a pregnant women reporting around 11-13 weeks for scan, the parameter of head circumference (HC) was added to FL and AC measurement. The average of these latter parameters inclusive of biparietal diameters are used to determine average gestational age and EDD. Placenta localization is also accurately determined. Those women with low lying placenta or suspected placenta praevia [21] had scans repeated subsequently especially at 34–36 weeks for placenta migration. Those who remained praevia were immediately sent to high level facility for management.

If danger signs were suspected, immediate actions were taken through the CHW, otherwise the feedback of the gynecologist was given to the woman a month after, when the ultrasound technician was coming for the new visit. Recognition of cases of abnormalities like ectopic pregnancies, fetal demise, missed abortion, incomplete abortion among others were immediately sent to appropriate facilities for management. Such patients were also followed up for recovery and counseling. Overall, the ultrasound scan acquisitions and examinations incorporate the WHO recommendations [22] and the protocols of Ghana. Data were stored in protected manner in a picture archiving and communication system.

### CHWs and ultrasound technicians training

The training of the CHWs was carried out with all CHWs together. Additionally, every month the ultrasound technicians while they were visiting the community were having short meetings to keep the motivation of CHWs high and to let them feel supervised and recognized [23, 24]. The CHWs were equipped with pregnancy test materials, uniforms, learning materials, mid-upper arm circumference measuring tapes, a mobile phone with the DocmeUp application, and a crank charger in case of limited access to electricity. No financial reward was given.

The trained ultrasound technicians were not medical doctors, but individuals with high level of education (MSc in Mathematics or Computer Science) with strong interest in Biomathematics/Biostatistics or Medicine. In this pilot only two technicians were involved. They were trained for several hours across three months under the supervision of an expert gynecologist, who trained them to acquire professionally obstetric ultrasound scans in order to be afterwards evaluated by official physicians. This was done to prove that the approach can be carried out even without the presence of a certified midwife or doctor within the community. The technicians were supported financially.

#### Ethical approval

The study has been approved by the local institutional review board of the Noguchi Memorial Institute for Medical Research, University of Ghana, Accra, Ghana. All participants received explanations regarding the purpose of the study and gave their written informed consent.

## Results

This section comprises the results of the survey to assess the initial baseline level, and the results obtained from the pilot study.

### Result of survey preceding roll out of pilot ANC system

The women interviewed were aged between 19 and 49, with a mean age (± standard deviation) of 29.4 (± 6.5) years. Among the 100 women, 80 at some time in their pregnancy experienced at least one of the complications listed in Table 1, and how the women handled these complications are displayed in Table 2.

**Table 2:** How women handled pregnancy complications reported in the survey (*n*=100, categories are not mutually exclusive)

How handled	Frequency
Visited health facility	63
Self-medication with traditional herbs	23
Did nothing	13
Self-medication with medicine bought from a drug store	11
Treated by traditional herbalist	5
Pray by religious leader as the only intervention	1
Other	1

Fifty-four (54) women had at least four ANC visits, 31 had one to three visits and 15 had no visit. Out of the 85 who had at least one visit, 27 (31.7 %) had their first visit during their first trimester, 44 (51.7 %) in the second trimester and 14 (16.6 %) in the third trimester. Reasons for no ANC visits were:

- insufficient money to cover cost involved, especially transportation cost (6 women, 40 %),
- uncertainty of being pregnant (4 women, 27 %),
- lack of knowledge about (importance of) ANC (2 women, 13 %),
- busy with work (2 women, 13 %),
- proximity to ANC clinic (1 woman, 7 %).

The reasons raised for late initiation of ANC were:

- uncertainty of being pregnant (24 women, 41 %),
- cost of ANC (19 women, 33 %),
- lack of knowledge of the importance of early ANC (14 women, 24 %),
- preferred not to answer (1 woman, 2 %).

Out of all 100 pregnancies, 81 resulted in live births, 7 ended in stillbirths (all of which were full term pregnancies), and 12 ended in miscarriages. Out of the 88 pregnancies that resulted in births, there were 29 deliveries (33 %) done at a health facility, 55 (62.5 %) were done at home and without a skilled attendant and 4 (4.5 %) were done at home but with a skilled attendant (thus, 33 representing 37.5 % of births were attended by skilled attendants and 55 representing 62.5 % were not). Reasons for home deliveries are listed in Table 3. Women who had their births attended by skilled attendants were less likely to have retained placenta than those who did not (Fisher’s Exact Test, two sided *p*−*value*=0.049, Pearson *χ*^2^, *p*−*value*=0.031). Women who attended ANC were less likely to have a miscarriage than those who did not (*χ*^2^, *p*−*value*<0.001). Women who attained the minimum recommended ANC visits were less likely to practice self-medication than those who did not. (*χ*^2^, *p*−*value*=0.03). The distribution of hospital deliveries and miscarriages according to community is reported in Table 4.

**Table 3:** Reasons for home deliveries reported in the survey (*n*=100)

Home delivery reason	Frequency
Cost involved in hospital delivery and transportation	29
to health facility	
Unavailability of transportation	18
No adequate preparation due to lack of knowledge of EDD	16
Fear of surgical operation	6
Dislike for health facility environment	5
Did not want medical intervention	4
Did not want male attendants	3
Previously had bad experience at health facility	3
Pressure from husband	1

**Table 4:** Distribution of hospital deliveries (*n*=88) and miscarriages (*n*=12) for women reported in the survey (*n*=100)

Community	Delivered at health facility	Delivered at home	Total delivery	Miscarriages
Biriwa	7	4	11	3
Ekumfi-Edumafa	13	25	38	4
Ekumfi-Asafa	7	21	28	5
Ankokrom-Owomasi	2	9	11	0
Total	29	59	88	12

The focus group discussion carried by the CHWs highlighted some dangerous misconceptions [25] and difficulties common among the women of the communities:

- *“When a woman experiences the swelling of hands or face, she does not believe that this might be a blood pressure dysfunction, but that she is expecting a boy”* (Ankokrom-Owomasi CHW).
- *“Many women believe that the swelling of hands and legs has to do with their salt intake, and they have just to reduce it”*(Ekumfi-Asafa CHW).
- *“It is difficult to explain family-planning, because for certain people, having a big family is instead their major pride.”*(Ekumfi-Edumafa CHW).

A topic that appeared commonly was the pre-eclampsia disorder and transportation issues.

### Pilot project results

From April 2014 to February 2015, the health workers followed 323 new pregnancies, sending 662 forms using the mobile phone, 323 of them to register pregnancies and 339 to inquire about danger signs. The distribution of the followed women and reported signs across the communities is summarized in Table 5. Not all women of the community accepted to be enrolled in the study. The reasons they refused to be enrolled were related to pressure of their husband or the local traditional herbalist. The distribution of ANC attendance and hospital deliveries for women who gave birth during the pilot project is summarized respectively in Tables 6 and 7.

**Table 5:** Pregnant women involved in each community during the pilot (*n*=323)

Community	Followed women	Danger signs
Biriwa	202	318
Ekumfi-Edumafa	81	46
Ekumfi-Asafa	37	21
Ankokrom-Owomasi	13	24

**Table 6:** Distribution of ANC attendance for women who gave birth during the pilot project (*n*=40)

Community	0 visit	1-3 visits	More than 3 visits
Biriwa	0	2	11
Ekumfi-Edumafa	0	2	8
Ekumfi-Asafa	0	3	6
Ankokrom-Owomasi	1	2	5
Total	1 (2 %)	9 (23 %)	30 (75 %)

**Table 7:** Distribution of Hospital Deliveries for Women who gave birth during the pilot project (*n*=40)

Community	Delivered at	Delivered	Total
	health facility	at home	
Biriwa	9	4	13
Ekumfi-Edumafa	7	3	10
Ekumfi-Asafa	6	3	9
Ankokrom-Owomasi	3	5	8
Total	25 (62.5 %)	15 (37.5 %)	40 (100 %)

Ultrasound technicians acquired ultrasound scans from (on the average) 30 pregnant women each month, among those who could not attend visits at the hospital. Ultrasound examination sessions were carried out on 122 women in all, including all the 60 women who gave birth during the period in exam. Some women had up to 4 examination sessions within the time they were on the project. One case of ectopic pregnancy was detected and early medical intervention was sought. Moreover, two women having breech presentation were detected and specially assisted to have their delivery attended by a professional birth attendant.

Between April 2014 to February 2015, 40 women who were on the project gave birth. Among the women on the project, 15 (37.5 %) delivered at home and 25 (62.5 %) delivered in a health facility.

The distresses of the women in the project reported by the CHWs are indicated in Table 8, where some pregnant women reported more than one sign. Following the counseling instruction of the mobile phone app, some women were encouraged to take Hepatitis B test and three tests came out positive. The tests were not performed by the CHWs.

**Table 8:** Danger signs reported to CHWs during the pilot (*n*=323)

Complication	Frequency
Persistent dizziness or headaches	107
Heart palpitations	36
Blurred vision	27
Fever	4
Severe nausea and vomiting	44
Recurring pain in abdomen or chest	80
Swelling of face and/or leg	14
Vaginal bleeding	15
Decreased fetal movement	37
Severe diarrhea	6
Accident or impact	29
Fainting	4
Other	6

## Discussion

From data sent to the online platform by the CHWs using the mobile phones, it was possible to know the number of ANC visits by each pregnant woman and number of deliveries attended by professional/skilled birth attendants. This study reports some qualitative and quantitative results.

By comparing the results of the survey pre-pilot (*n*=100) and the dangerous signs reported during the pilot (*n*=323) some observations can be made. ”Persistent dizziness or headaches“ and ”recurring pain in the abdomen or chest“ are still present proportionally in the communities while other dangerous signs appear to be decreased. The number of ”accident or impact“ has instead increased, raising from 6 cases for 100 women of the survey to 29 for 323 women involved in the pilot. However, these comparisons have to take into account the different sample size and the recall bias as possible confounding factors.

Moreover, it was noted that the number of registered women from the Biriwa community reported by the CHW was larger than those reported from the other communities. This can be due to the larger population present in the community, which was difficult to assess. Another possible explanation is the factor that the CHW of the Biriwa community was more dynamic and active compared to the others in registering pregnant women.

Another qualitative observation was that even with the same level of education, younger CHWs learned the use of the mobile phone application faster than their older colleagues, which asked several times clarifications on the use of the mobile application. Therefore, age can be a further selection criteria for CHWs. Overall, it is worthwhile to mention that in the communities located less than 5 km from a clinic, most women interviewed in the preceding phase of the pilot study and during the pilot study delivered in hospitals or clinics. This suggests that the proposed approach is better targeted in rural communities distant more than 5 km from clinics.

The approach showed a statistically significant increase in ANC attendance and hospital deliveries comparing the results of the pilot with the data gathered in the pre-pilot survey. However, due to the several components of the project, it is hard to understand which component was the most relevant. It is believed that the ultrasound scans in the community help to detect the ectopic and breech pregnancy which otherwise would not be recognized in time, since the women were not attending proper ANC. However, the overall improved ANC could be attributed to the CHWs.

Ectopic pregnancy is a complication of pregnancy in which the embryo attaches outside the uterus [7]. The symptoms of ectopic pregnancies are abdominal pain and vaginal bleeding, but ultrasound examination is required to confirm it [7], and the proposed approach with portable ultrasound acquisitions can be a valid aid in this context. In fact, the case reported in this study was discovered by the ultrasound technician in a woman who was reporting vaginal bleeding and had no further suspicion. In a breech birth, the baby exits the pelvis with the buttocks or feet first as opposed to the normal head-first presentation and this can stem some complications. The discovery of “breech pregnancies” is more challenging without the use of ultrasound, since the only sign is the eventual kicking of the baby after 36 weeks or the examination of a skilled midwife [6]. In the reported pilot, these have been discovered by the ultrasound technicians during their examination while the women were not aware of the associated risks. It is worthwhile to mention qualitatively that both the CHWs and the ultrasound technicians revealed how delighted the project beneficiaries (pregnant women) felt about having ultrasound acquisitions in their own community.

Hypothesizing the expansion across Ghana as a whole, at least one ultrasound technician equipped with portable ultrasound per district will be needed, therefore approximatively at least 216 ultrasound technicians will be required, though more technicians might be required for densely populated areas. A CHW for an average of 500 people in rural communities should be employed, with a resulting need of 35.000 CHWs, which is in accordance with the goals of the *”One Million Community Health Workers Campaign“* in Ghana [26]. In fact, in order to reach the health MDGs, Ghana’s Ministry of Health has already partnered with the *One Million Community Health Workers Campaign* to strengthen the existing community-based Health Planning and Service program through the addition of CHWs [26]. Moreover, the proposed framework can be integrated into mobile health policies currently running in Ghana like MoTech [27], although a cost-benefit analysis [28] needs to be carried out. Alternatively, once the importance of the delivery at hospital has been understood and behavioral change occurred [29], the pregnant women of each community could pool informally some capital to finance the expenses and allowance of the ultrasound technician [30]. In addition, it has been highlighted that projects promoting ANC require also the availability of reliable transportation means the pregnant women at the time of labor [30]. The main limitation of the study is the sample size due to resource constraints. Rather than the number of women involved, the limitation may concern the limited number of communities which cannot depict the large variability which can be encountered in the whole country. Another limitation can be the recall bias which the women interviewed during the survey preceding the roll out of the pilot. Therefore these confounding factors have to be borne in mind in the comparison between the danger sings reported in the survey and the pilot.

It has been preferred to endow only the CHWs with mobile phones since the focus was to promote professional figures within the communities in a way that can be easily scaled, similarly to study previously carried out in Rwanda called RapidSMS-MCH [13], where SMS alerts were used by CHWs for urgent and life threatening events. A two-way communications using CHWs and mothers has been already used in two projects, the Wired-Mothers project in Tanzania [18] and MoTech in Ghana [27]. Where both projects aimed at increasing the proportion of women attending the recommended ANC visits by sending direct reminder SMS to the pregnant women and by coordinating the CHWs. Despite the approach is considered valuable, it is beyond the purpose of the pilot which was more about integrating ultrasound scan acquisitions with community initiatives, and about the possibility of using ultrasound technicians who were not medical doctors nor received a full education as sonographers.

Future work includes the use of automatic delineation of biometric features of the foetus and regression models on the acquired ultrasound scans, since these algorithms will estimate automatically the delivery date and current weight of the fetus which will ease even more the amount of work to the gynecologist [31].

## Conclusion

The pilot project described in this manuscript showed promising results by using an application for low-cost mobile phones and portable ultrasound machines, by offering professional monitoring and supervision in rural areas where ANC was previously difficult. Mobile phones and portable ultrasound machines can help women and local health workers to seek appropriate medical help. These tools allow early detection of specific adversities - such as the detected ectopic and breech pregnancies - which is likely to reduce the time between they appear and when professional care is sought.

## Abbreviations

ANC: ante-natal care
MDG: millennium development goal
CHW: community health worker
SMS: short messaging service
IT: information technology
GPRS: general packet radio service
EDD: estimated delivery date
FL: femur length
AC: abdominal circumference
HC: head circumference
